# Molecular Detection of *Borrelia burgdorferi* Sensu Lato and *Anaplasma phagocytophilum* in Ticks Collected from Dogs in Urban Areas of North-Eastern Poland

**DOI:** 10.3390/pathogens9060455

**Published:** 2020-06-09

**Authors:** Mirosław M. Michalski, Katarzyna Kubiak, Magdalena Szczotko, Marta Chajęcka, Małgorzata Dmitryjuk

**Affiliations:** 1 Department of Parasitology and Invasive Diseases, Faculty of Veterinary Medicine, University of Warmia and Mazury in Olsztyn, Oczapowskiego 13, 10-719 Olsztyn, Poland; michmm@uwm.edu.pl; 2 Department of Medical Biology, Collegium Medicum, School of Public Health, University of Warmia and Mazury in Olsztyn, Zolnierska 14c, 10-561 Olsztyn, Poland; katarzyna.kubiak@uwm.edu.pl; 3 Department of Biochemistry, Faculty of Biology and Biotechnology, University of Warmia and Mazury in Olsztyn, Oczapowskiego 1A, 10-719 Olsztyn, Poland; magdalena.szczotko@uwm.edu.pl (M.S.); marta.chajecka@student.uwm.edu.pl (M.C.)

**Keywords:** *Borrelia burgdorferi* sensu lato, *Anaplasma phagocytophilum*, *Ixodes ricinus*, *Dermacentor reticulatus*, ticks, dogs, urban areas

## Abstract

From 2016 to 2018, ticks were collected from 272 dogs admitted to veterinary clinics in the city of Olsztyn (north-eastern Poland). Among 522 collected ticks, 423 were identified as *Ixodes ricinus* (413 females and 10 males) and 99 as *Dermacentor reticulatus* (62 females and 37 males). Non-engorged (86 individuals) and engorged (436 individuals) ticks were screened for the presence of *Borrelia burgdorferi* sensu lato and *Anaplasma phagocytophilum* DNA. *Borrelia* and *A. phagocytophilum* species detection was determined based on the sequence of the *fla B* and *16S RNA* genes, respectively. DNA of *B. burgdorferi* s.l. was identified in 31.6% (165/522, 95% CI: 27.6–35.8%) of ticks (*I. ricinus* 151/423, 35.7%, 95% CI: 31.1–40.4%; *D. reticulates* 14/99, 14.1%, 95% CI: 7.9–22.6%). *A. phagocytophilum* was identified in 0.96% (5/522, 95% CI: 0.3–2.2%) of specimens. All positive samples were engorged *I. ricinus* females (5/402, 1.2%, 95% CI: 0.4–2.9%). In 85.4% (141/165, 95% CI: 79.1–90.4%) of *Borrelia* infected ticks, the DNA of one genospecies was revealed. The DNA of at least two different genospecies was detected in 14.5% of specimens (24/165, 95% CI: 9.5–20.8). The coexistence of *B. burgdorferii* s.l. and *A. phagocytophilum* was not detected.

## 1. Introduction

Along with mosquitoes, ticks are the most widespread vectors of pathogenic microorganisms (viruses, bacteria, and protozoa) for humans and domestic animals worldwide [1,2,3]. *Ixodes ricinus* and *Dermacentor reticulatus* are the most important tick species in Poland, but the role of *I. ricinus* in pathogen transmission is dominant [4]. One of the most frequently diagnosed zoonotic tick-borne diseases is Lyme borreliosis (LB). Worldwide, the main vectors of the *B. burgdorferi* s.l. are *I. ricinus*, *I. scapularis*, and *I. persulcatus*. In Central Europe, *I. ricinus* represents the main health risk to humans and many other vertebrate species as a vector of multiple pathogens, including *Borrelia* spirochetes [5]. The pathogens causing LB are spirochetes included in the *Borrelia burgdorferi* complex, which now comprises ca 20 *Borrelia* species. Nine of them have been detected in European *I. ricinus* ticks. The most common genospecies of *Borrelia* in Europe are *B. afzelii*, *B. garinii*, *B. burgdorferii* sensu stricto (*B. burgdorferi* s.s.), *B. valaisiana*, and *B. lusitaniae* [6]. Three of these genospecies (*B. garinii*, *B. afzelii*, and *B. burgdorferi* s.s.) are clearly pathogenic to humans [7,8,9]. These species differ in organotropism and they cause different LB clinical symptoms in humans: *B. afzelii* is mainly associated with skin manifestations of LB-migratory erythema (EM) and chronic atrophic dermatitis (ACA), *B. burgdorferi* s.s. with changes in the osteoarticular system, and *B. garinii* with neurological symptoms [9].

The reservoirs of the *B. burgdorferi* spirochetes are rodents, medium-sized mammals (mainly from the Cervidae and Canidae families), birds, and lizards [10,11]. Domestic and farm animals often undergo a mild, usually undiagnosed, form of LB. Clinical LB caused by *B. burgdorferi* s.s. has nonetheless been reported in dogs, horses, and cats [12,13]. Domestic and wild animals usually play a passive role in the epizootic chain by transmitting ticks, the main vector of infection. Most often, wild animals are a reservoir of *B. burgdorferi* and they themselves show a tolerance to this bacterium. They do not get sick, but they are the source of infection for feeding ticks [11].

Anaplasmosis is a zoonotic multi-organ disease of humans and animals. This disease is caused by *Anaplasma phagocytophilum*. It is an obligatory intracellular, Gram-negative bacterium that mainly inhabits the granulocytes of peripheral blood [14,15]. *I. ricinus* is the only known vector for *A. phagocytophilum* in Central Europe [16,17]. Reservoir animals for *A. phagocytophilum* are predominantly roe deer, livestock (cattle, sheep, horses), small rodents (mice, shrews, voles), and pet animals, mainly dogs [14]. Human granulocytic anaplasmosis (HGA) may occur in the absence of associated clinical signs, and cases may not always be detected. In symptomatic patients, most present with fever, headache, fatigue or malaise, myalgia, arthralgia, and nausea. Other clinical observations in humans have included renal, pulmonary, and neurological complications, which may be accompanied by thrombocytopenia, leukopenia, and normocytic anemia [18,19]. *A. phagocytophilum* may cause canine granulocytic anaplasmosis (CGA) [20]. Most dogs naturally infected with this pathogen show no symptoms of the disease, despite serological evidence of infection [14]. After an incubation period of 1–2 weeks, the most common clinical signs are lethargy and fever. Less commonly reported symptoms also include coughing, diarrhea, anorexia, reluctance to move, lameness, enlargement of lymph nodes, pale mucous membranes, and hemorrhage [14,15,20,21,22,23].

Recreational green areas within city agglomerations could be a favorable habitat for ticks and their hosts [24,25,26]. In these areas, tick hosts, i.e., reservoirs of pathogens and primary sources of infection, are mainly small mammals (rodents, hedgehogs, squirrels) and birds [26,27]. A similar role of pets (dogs and cats) is highly probable [26,28,29]. In Poland, 30% of city residents declare owning a dog (in Olsztyn there are currently about 9000 dogs) [30], which can be parasitized by five tick species: *I. ricinus*, *I. hexagonus*, *D. reticulatus, I. crenulatus*, and *I. rugicollis* [28,31]. This justifies the regular prophylactic screening of dogs for tick infestation and for tick-borne diseases. In addition, the collection of ticks from companion animals combined with a screening for tick-borne pathogens can provide information about the potential infection risk for people [27].

*I. ricinus* has a three-host life cycle. Before it molts, it ingests the blood of another host in each life stage. In the case of *Borrelia* spp. and *A. phagocytophilum*, transovarial transmission of a pathogen is very rare and DNA detection of these agents in feeding larvae is proof of pathogen transmission from an infected reservoir host to the tick [15,32,33]. Therefore, pathogens have the ability to persist throughout the molting process to the next developmental stage of tick vectors by transstadial transmission [13]. The most numerous tick species isolated from dogs in Europe is *I. ricinus* [25,34]. Many studies have shown that adult ticks are significantly more infected with spirochetes and *A. phagocytophilum* than nymphs [5,35,36,37,38]. Additionally, dogs can be useful for collecting ticks in a way similar to flagging, and the prevalence of infection in ticks removed from dogs provides an estimate of the risk of dogs becoming infected by tick-borne disease agents [38].

*D. reticulatus*, the second most abundant tick species in many parts of Europe after *I. ricinus*, can transmit to a host the protozoa *Babesia canis*, bacteria from the *Rickettsia* and *Anaplasma* genera, or the tick-borne encephalitis virus [39]. The participation of *D. reticulatus* in the transmission of *B. burgdorferi* s.l. is still pending, although the specific DNA of this pathogen has been detected in these ticks [40,41]. However, there is still no evidence of its role as a vector of spirochetes [4].

Based on the “One Health” theory, tick-borne diseases are associated with close relationships among ticks, humans, and companion animals. Additionally, the monitoring of tick-borne pathogens in ticks attached to animals is important to determine disease distribution and possible transmission to humans [42]. Considering the information above, the aim of this study was the detection of *B. burgdorferi* s.l. and *A. phagocytophilum* in non-engorged and engorged *I. ricinus* and *D. reticulatus* ticks collected from dogs in the urban areas of north-eastern Poland, a region endemic for tick-borne diseases.

## 2. Results

### 2.1. Tick Collection

Vets from three veterinary clinics in Olsztyn collected non-engorged and engorged ticks from dogs between spring 2016 and autumn 2018. A total of 522 adult ticks were identified: 81% *I. ricinus* (423/522—413 females and 10 males) and 19% *D. reticulatus* (99/522—62 females and 37 males). All of the ticks were removed from a total of 272 dogs (on average there were 1.92 ticks per dog). These ticks were stored at −4 °C for further analysis.

### 2.2. Molecular Identification of Pathogens

Overall, 522 adult ticks were analyzed (Table 1). *B. burgdorferi* DNA was detected in 165 (31.6%, 95% CI: 27.6–35.8%) ticks. Statistically significant differences (χ^2^ = 350.2, *p* < 0.05) were noted between the mean tick infections in *I. ricinus* (151/423, 35.7%, 95% CI: 31.1–40.4%) and *D. reticulatus* (14/99, 14.1%, 95% CI: 7.9–22.6%, Table 1). The percentage of *Borrelia*-positive ticks was significantly higher statistically in engorged ticks in comparison to non-engorged ticks (153/436, 35.1%, 95% CI: 30.6–39.8%; 12/86, 13.9%, 95% CI: 7.4–23.1%, respectively; χ^2^ = 14.85, *p* < 0.05, Table 1). In 85.4% (141/165, 95% CI: 79.1–90.4%) of *Borrelia*-positive ticks, the DNA of one genospecies was revealed. The DNA of at least two different genospecies was detected in 14.5% of specimens (24/165, 95% CI: 9.5–20.8%). *B. garinii* was the predominant mono-infective species (114/141, 80.9%, 95% CI: 73.4–86.9%), while the less numerous species were *B. afzelii* (14/141, 9.9%, 95% CI: 5.5–16.1%) and *B. burgdorferi* s.s. (13/141, 9.2%, 95% CI: 5.0–15.2%). The results were significant at *p* < 0.05 for the occurrence of *B. garinii* and the other two genospecies (χ^2^ = 214.91). *B. garinii* and *B. afzelii* co-occurred most often (12/24, 50%, 95% CI: 29.1–70.9%). *B. afzelii/B. burgdorferi* s.s (6/24, 25%, 95% CI: 9.7–46.7%) and *B. garinii/B. burgdorferi* s.s. (5/24, 20.8%, 95% CI: 7.1–42.1%) co-occurred less frequently. There was only one case with a coinfection of all three pathogens (1/24, 4.2%, 95% CI: 0.1–21.1%; Table 1). *A. phagocytophilum* was identified in 0.96% (5/522, 95% CI: 0.3–2.2%) of specimens. All positive samples were *I. ricinus* (5/423, 1.2%, 95% CI: 0.4–2.9%; Table 1). Coinfections with. *A. phagocytophilum* and *B. burgdorferii* s.l were not detected.

### 2.3. BLASTn Data Analysis

Comparative analysis with data registered in the GenBank database using BLASTn showed that all obtained sequences (Aph1-5: KY319143, KY828226, MK530241-MK530243) belonged to one haplotype and revealed 100% homology to the sequence of *A. phagocytophilum* first described in a human patient from Wisconsin, USA (U02521) and the *A. phagocytophilum* sequence from a tick removed from human skin in north-eastern Poland (DQ006828).

## 3. Discussion

Tick infestation and the risk of tick-borne diseases are commonly connected with forested and rural areas. However, many reports indicate that ticks are well adapted to urban and suburban environments [24,26,43,44]. Ticks that inhabit urban localities originate from tick populations persisting in wild natural habitats around cities and towns, and environmental conditions in both localities, urban and natural, are suitable and promote the development of a tick population [45,46]. In our opinion, in green areas located within the administrative boundaries of large cites, not only are residents exposed to the ticks and the pathogens transmitted by them, but so are their pets, such as domestic dogs and cats.

Lyme disease also has veterinary importance, affecting dogs, cattle, horses, and cats. The most common clinical signs in domestic animals are lameness, loss of appetite, weight loss, and kidney disease [47]. The infestation rates of domestic animals with pathogen-infected ticks are poorly documented in Europe, including in Poland. Even fewer publications report on the proportions of pathogen-infected ticks removed from animal or human skin. As early as the 1990s, in Germany (North Baden), 22% of *I. ricinus* and *I. hexagonus* removed from domestic animals were infected by *B. burgdorferi*, while in Lower Saxony, fewer than 10% of all human skin-attached ticks were *Borrelia*-positive. The PCR method was used in both studies using *23S rRNA* and *16S rRNA* genes, respectively [48,49]. In the latest research from Germany (2013–2017), the overall *Borrelia* infection rate of *I. ricinus* ticks attached to human skin by real-time PCR was 20.02% [50]. In 2016 in Great Britain, molecular analysis of the *ospA* gene confirmed that 1.8% of ticks collected from cat skin were *Borrelia*-positive, and the most frequent species was *B. garinii* [51]. In 2018 Geurden et al. [43] analyzed the prevalence of *Borrelia* spp. and *A. phagocytophilum* by the real-time PCR method. Ticks were collected from dogs and cats in Hungary, France, Italy, Belgium, and Germany. *Borrelia* spp. were mainly identified in *I. ricinus* collected from cats (18%) and to a lesser extent in dog-sourced ticks (1%); while *A. phagocytophilum* was also found in 17% of ticks. In Poland, a study was conducted on ticks collected in 2013 from domestic dogs and cats in the Wroclaw Agglomeration (south-west Poland). Using nPCR, the authors revealed that 22.5% and 21.3% of the ticks were *Borrelia*-positive, respectively. A comparable level of *Borrelia* infection between *I. ricinus* from pets and vegetation indicates that domestic animals may participate in the circulation of these pathogens, and that they do not have zooprophylactic competence [28]. Moreover, it is very well documented that there is a possibility of cross-infection (including *B. burgdorferi* and *A. phagocytophilum*) when multiple ticks, infected and non- infected specimens, are co-feeding on one animal [52].

We assumed that adult feeding ticks are characterized by the highest degree of infection due to the transstadial transmission of pathogens. It is known that engorged ticks cause problems in molecular testing. Substances present in mammalian blood can inhibit the PCR amplification. Beichel et al. [48] pointed out that ticks larger than 4 mm inhibit the PCR reaction. Therefore, in our study, only the anterior parts of engorged ticks were analyzed. We have shown that from 165 cases of *Borrelia* spp., as many as 153 cases were from engorged ticks and only 12 came from non-engorged ticks. The study of Scott et al. [8] showed that 36% of *I. scapularis* adults collected from 41 mammalian hosts (dogs, cats, humans) were positive for the Lyme disease bacterium. Actually, 35.7% of the examined *I. ricinus* and 14.1% *D. reticulatus* collected in the urban area of Olsztyn tested positive for the DNA of *Borrelia* spp. spirochetes. The dominant species of *B. garinii* was detected in over 80% of cases. In some contrast to our results, Kubiak et al. [44] showed that the overall infection rate of questing *I. ricinus* with *Borrelia* spirochetes was 27.4% in Olsztyn, and the dominant genospecies was *B. afzelii* (93.1%). It is noteworthy that they collected ticks by flagging while we specifically studied the ticks that were actively parasitizing dogs. Perhaps dogs in Olsztyn are mainly a reservoir of *B. garinii*, but this should be confirmed by a city-wide dog blood test for the presence of DNA from the *Borrelia* genospecies. This may be due to the different mechanism of transmission of the spirochete genospecies. Hovius et al. [38] observed a higher infestation of *B. burgdorferi* s.s. in non-engorged (either questing or attached) *I. ricinus* (12%) compared to semi-engorged ticks (2%), explaining this phenomenon by loss of infectivity during the start of the feeding phase. According to De la Fuente et al. [53], pathogen transmission by ticks requires many often unexplored tick–pathogen interactions, from the migration of these pathogens from the gut to their secretion in tick saliva. It is possible that *B. garinii* is not transmitted as quickly as the other two genospecies during tick feeding.

HGA, a zoonotic acute febrile disease, is difficult to recognize because its symptoms are rather nonspecific. European studies warn that the infection, although largely unrecognized, may be widespread in most of Europe, because the pathogen incidence in tick-carriers ranges from moderate to high, with the median prevalence of *A. phagocytophilum* in European *I. ricinus* ticks at approximately 3% [18,54,55]. In our study, only 5 out of 402 (1.2%) engorged *I. ricinus* females showed the presence of *A. phagocytophilum* DNA. The prevalence of *A. phagocytophilum* was 6% in the DNA of adult *I. ricinus* ticks collected from dogs in Latvia, whereas in *I. persulcatus* and *D. reticulatus*, the pathogen was not identified [56]. Even though similar studies are scarce, our data did seem rather low for the country. This is because in the urban areas of Wroclaw, 21.3% *I. ricinus* and 8.1% *I. hexagonus* ticks isolated from dogs and cats were *A. phagocytophilum*-positive [29]. This was higher than our rates of tick infection with *A. phagocytophilum* from 8.7% to 16.0%. These were also recorded in the north-eastern regions of Poland at the beginning of the century, and an extremely rapid increase was observed over the next few years of research, in some locations from 6.6% to a stunning 73.3% [37]. It is also important to note that in Poland, *A. phagocytophilum* more frequently infects ticks in urban areas than in natural forests [57]. Similar findings were also reported by Grzeszczuk and Stańczak [58] who published extremely disturbing data that 23.7% of *A. phagocytophilum*-positive *I. ricinus* ticks were removed from the skin of Bialystok residents (north-eastern Poland).

Nucleotide analysis of the partial sequence of the *Anaplasma 16S rRNA* gene showed that all amplified sequences belong to one haplotype. BLASTn analysis revealed a 100% similarity with the sequence of *A. phagocytophilum* from the first described human patient in Wisconsin (USA) and also with *A. phagocytophilum* from ticks removed from human skin in north-eastern Poland [58,59]. These results indicate that the Aph1-5 haplotype detected in *I. ricinus* collected from dogs in Olsztyn represents *A. phagocytophilum*, and that HGA in urban dogs may be significant in the future.

We also addressed the coexistence of *B. burgdorferii* s.l. and *A. phagocytophilum* in ticks, because in Pomerania (Pomorze, northern Poland) 5% of examined *I. ricinus* ticks contained both pathogens. Moreover, research in the urban and suburban forests in the Tri-City area of Gdańsk, Gdynia, and Sopot (northern Poland) showed the simultaneous presence of both pathogens in 8.3% of adult ticks. It is possible that *B. burgdorferii* s.l. and *A. phagocytophilum* consolidate in the same foci and often co-infect the same tick vector, thus increasing the risk of a mixed infection [60,61]. At the present time in Olsztyn, we have not observed such a dependence, most likely due to the low incidence (0.96%) of *A. phagocytophilum*. However, the epidemiological situation should be closely monitored, especially in the areas considered as endemic for tick-borne diseases.

## 4. Materials and Methods

### 4.1. Study Area and Tick Collection

For the analysis we used ticks collected from domestic dogs visiting veterinary clinics in the Olsztyn agglomeration (53°47′ N 20°29′ E; 88.33 km^2^; 173,070 inhabitants) between 2016 and 2018. Olsztyn is the capital of the Warmia-Masuria Province in north-eastern Poland. Within the city there are a large number of parks, squares, and forested recreational areas, which occupy a quarter of the city. These areas also provide an excellent habitat for ticks. Ticks were collected from the dogs by veterinarians and were placed in tubes with 70% ethanol. In the laboratory, the species and sex of the ticks were identified, then, individuals were weighed, measured, and separated into non-engorged and engorged categories. To the non-engorged *I. ricinus* female group, those weighing on average 2.13 ± 1.15 mg and measuring 3.63 ± 0.50 mm were included; while the engorged group weighed on average 104.26 ± 112.29 mg and measured 7.84 ± 2.36 mm. Non-engorged *I. ricinus* males weighed and measured 0.94 ± 0.2 mg and 2.7 ± 0.48 mm accordingly, while non-engorged *D. reticulatus* included individuals weighing on average 4.94 ± 0.98 mg and 4.69 ± 1.45 mg, and measuring 4.64 ± 0.49 mm and 4.38 ± 0.46 mm, for females and males, respectively. Those classified as *D. reticulatus* engorged females were the largest of the analyzed ticks. They weighed and measured on average 220.29 ± 140.65 mg and 11.2 ± 2.68 mm.

### 4.2. DNA Extraction

To start, the ticks preserved in 70% ethanol were dried. Fully-engorged ticks were bisected, only the anterior end was used. Anterior parts or entire ticks were then crushed using a sterile mortar, moved to 2 mL tubes filled with lysis buffer (A&A Biotechnology, Gdynia, Poland) and were incubated for 2 h at 50 °C. Total DNA was extracted according to the manufacturer’s tissue protocol (Micro AX Tissue Gravity, A&A Biotechnology, Gdynia, Poland) and stored at −70 °C.

### 4.3. PCR Conditions

The *Borrelia* region of the *fla B* gene (422 bp) was amplified using the primer BFL1/BFL2 [62] (Table 2) and PCR was run under the following thermal cycle conditions: an initial activation step of 2 min at 94 °C was followed by 40 cycles of 30 s at 94 °C, 1 min at 58 °C, and 1 min at 72 °C. Finally, an extension step of 1 min was performed at 72 °C. For *A. phagocytophilum*, the primer sets EHR521 and EHR747 [63] (Table 2) were used to amplify a 247 bp fragment from the *16S RNA* gene under the following thermal cycle conditions: an initial activation step of 5 min at 94 °C was followed by 40 cycles of 45 s at 94 °C, 45 s at 54 °C, 45 s at 72 °C, and a final extension of 5 min at 72 °C. In both cases, 5 μL of the DNA extracted from each tick was added to 20 μL of reaction mixture comprised of 12.5 μL DreamTaq Green PCR Master MIX (Thermo Scientific, Waltham, MA, USA), 9.4 μL nuclease-free water, and 0.05 μL of each primer (100 μM). All reactions were carried out using a Mastercycler Nexus (Eppendorf, Hamburg, Germany). PCR products were visualized by electrophoresis on 1.5% agarose gel stained with Midori Green DNA dye (Nippon Genetics Europe GmbH, Düren, Germany). Positive tick samples were analyzed twice. Each PCR analysis included negative (nuclease-free water instead of DNA) and positive control samples. The positive control was commercial DNA from *B. burgdorferii* s.l. purchased from the DNA Gdańsk Company (Gdańsk, Poland). Positive control samples for the *A. phagocytophilum* 16S rRNA gene included purified and confirmed samples obtained by sequencing genomic DNA from previously positive samples.

### 4.4. PCR-RFLP Analysis

PCR-RFLP analysis was carried out to identify three genospecies containing the *B. burgdorferi* s.l. complex: *B. garinii*, *B. afzelii*, and *B. burgdorferi* s.s. The amplified DNA was digested with the *TasI* endonuclease (Fast Digest Tsp 509I, Thermo Fisher Scientific, Waltham, MA, USA) to obtain the restriction patterns of the different genospecies of the spirochetes. For each positive sample, 10 μL of amplified DNA were digested in a Mastercycler Nexus (Eppendorf, Hamburg, Germany) at 65 °C for 15 min. The reaction mixture contained 17 μL of nuclease-free water, 2 μL of 10 × Fast-Digest buffer, and 1 μL of *TasI* enzyme. PCR-RFLP products were separated by electrophoresis in a 3% agarose gel and stained with Midori Green dye. DNA fragments subjected to restriction analysis were visualized in the G-BOX Syngene transilluminator. Membership in a given genospecies was determined by *TasI* restriction fragment sizes (bp) according to restriction patterns (Table 2) [62].

### 4.5. DNA Sequencing and Data Analysis

PCR products of the positive *Anaplasma* samples and the chosen positive products for *Borrelia* spirochetes were purified using the Clean Up purification kit (A&A Biotechnology, Gdynia, Poland) according to the manufacturer’s protocol and bidirectionally sequenced at Genomed (Warsaw, Poland). The obtained nucleotide sequences were assembled and compared with data registered in the GenBank database using BLASTn (https://blast.ncbi.nlm.nih.gov) to confirm the attachment to the *A. phagocytophilum* or *B. burgdorferi* s. l. complex. The consensus *A. phagocytophilum 16S rRNA* gene and the *Borrelia fla* gene sequences were deposited in the GenBank database and registered under the access numbers KY319143, KY828226, MK530241-MK530243 (Aph1-5; *A. phagocytophilum 16 S rRNA subunit* gene), MK834321-MK834322 (*Borrelia* sp. *fla* gene—*B. garinii*), MK834319-MK834320 (*Borrelia* sp. *fla* gene—*B. afzelii*), and MK834317-MK834318 (*Borrelia* sp. *fla* gene—*B. burgdorferi* s.s.).

### 4.6. Statistical Analysis

All ticks were analyzed individually, and the prevalence was expressed as a percentage. Statistical analysis of the results was carried out using the two-sided Fisher’s exact test (Prism 7 program, GraphPad Software, San Diego, CA, USA). Prevalence of pathogens was calculated with 95% confidence intervals (95% CI). In order to check whether there was a prevalence of *Borrelia* in both tested tick species or non-engorged and engorged ticks and a relationship between variables (the occurrence of *B. garinii* and the other two genospecies), the Chi-square test (χ^2^) was used. Values of *p* < 0.05 were considered statistically significant.

## 5. Conclusions

This study indicates that domestic dogs in north-eastern Poland are at risk of infection from *Borrelia* species and *A. phagocytophilum*. The results of this study demonstrate the potential danger from ticks feeding on dogs. Ticks represent a serious risk of LB for companion animals and for city residents. There is a high percentage of *Borrelia*-positive ticks in the city of Olsztyn (north-eastern Poland); *A. phagocytophilum*-positive ticks were identified less frequently. However, veterinarians and physicians should be aware of anaplasmosis among domestic animals and among patients with a tick-bite history.

## Figures and Tables

**Table 1:** Infection rates of *Ixodes ricinus* and *Dermacentor reticulatus* ticks removed from dogs in the Olsztyn-city agglomeration, north-eastern Poland, with the *Borrelia* genospecies and *Anaplasma phagocytophilum*.

	*Ixodes ricinus*			*Dermacentor reticulatus*			
Pathogens	* NE Females (%) (95% CI)	** E Females (%) (95% CI)	* NE Males (%) (95% CI)	* NE Females (%) (95% CI)	** E Females (%) (95% CI)	* NE Males (%) (95% CI)	Total Ticks (%) (95% CI)
*B. garinii*	0/11 (0.0)	113/402 (28.1)	0/10 (0.0)	0/28 (0.0)	0/34 (0.0)	1/37 (2.7)	^3^ 114/141 (80.9)
	(0.0–28.5)	(23.8–32.8)	(0.0–30.9)	(0.0–12.3)	(0.0–10.3)	(0.07–14.1)	(73.4–86.9)
*B. afzelii*	1/11 (9.1)	13/402 (3.2)	0/10 (0.0)	0/28 (0.0)	0/34 (0.0)	0/37 (0.0)	^3^ 14/141 (9.9)
	(0.2–41.3)	(1.7–5.4)	(0.0–30.9)	(0.0–12.3)	(0.0–10.3)	(0.0–9.5)	(5.5–16.1)
*B. burgdorferi* s.s.	0/11 (0.0)	8/402 (2.0)	0/10 (0.0)	0/28 (0.0)	0/34 (0.0)	5/37 (13.5)	^3^ 13/141 (9.2)
	(0.0–28.5)	(0.8–3.9)	(0.0–30.9)	(0.0–12.3)	(0.0–10.3)	(4.5–28.8)	(5.0–15.2)
*Borrelia* Monoinfections	1/11 (9.1)	134/402 (33.3)	0/10 (0.0)	0/28 (0.0)	0/34 (0.0)	6/37 (16.2)	141/165 (85.4)
	(0.2–41.3)	(28.7–38.2)	(0.0–30.9)	(0.0–12.3)	(0.0–10.3)	(6.2–32.0)	(79.1–90.4)
*B. garini*/*B. afzelii*	0/11 (0.0)	9/402 (2.2)	0/10 (0.0)	0/28 (0.0)	3/34 (8.8)	0/37 (0.0)	12/24 (50.0)
	(0.0–28.5)	(1.0–4.2)	(0.0–30.9)	(0.0–12.3)	(1.8–23.7)	(0.0–9.5)	(29.1–70.9)
*B. garinii/B.burgdorferi* s.s.	0/11 (0.0)	3/402 (0.75)	0/10 (0.0)	0/28 (0.0)	3/34 (8.8)	2/37 (5.4)	5/24 (20.8)
	(0.0–28.5)	(0.15–2.1)	(0.0–30.9)	(0.0–12.3)	(1.8–23.7)	(0.6–18.2)	(7.1–42.1)
*B. afzelii/B. burgdorferi* s.s.	0/11 (0.0)	3/402 (0.75)	0/10 (0.0)	0/28 (0.0)	0/34 (0.0)	3/37 (8.1)	6/24 (25.0)
	(0.0–28.5)	(0.15–2.1)	(0.0–30.9)	(0.0–12.3)	(0.0–10.3)	(1.7–21.9)	(9.7–46.7)
*B. garinii/B. afzelii/B. burgdorferii* s.s.	0/11 (0.0)	1/402 (0.25)	0/10 (0.0)	0/28 (0.0)	0/34 (0.0)	0/37 (0.0)	1/24 (4.1)
	(0.0–28.5)	(0.006–1.4)	(0.0–30.9)	(0.0–12.3)	(0.0–10.3)	(0.0–9.5)	(0.1–21.1)
*Borrelia* Coinfections	0/11 (0.0)	16/402 (4.0)	0/10 (0.0)	0/28 (0.0)	3/34 (8.8)	5/37 (13.5)	24/165 (14.5)
	(0.0–28.5)	(2.3–6.4)	(0.0–30.9)	(0.0–12.3)	(1.8–23.7)	(4.5–28.8)	(9.5–20.8)
*Borrelia* Total	^1^ 151/423 (35.7)			^1^ 14/99 (14.1)			165/522 (31.6)
	(31.1–40.4)			(7.9–22.6)			(27.6–35.8)
*Borrelia* Total ** E	150/436 (34.4)			3/436 (0.7)			^2^ 153/436 (35.1)
	(29.9–39.1)			(0.14–2.0)			(30.6–39.8)
*Borrelia* Total * NE	1/21 (4.7)			11/65 (16.9)			^2^ 12/86 (13.9)
	(0.12–23.8)			(8.7–28.2)			(7.4–23.1)
*A. phagocytophilum*	0/11 (0.0) (0.0–28.5)	5/402 (1.2) (0.4–2.9)	0/10 (0.0) (0.0–30.9)	0/28 (0.0) (0.0–12.3)	0/34 (0.0) (0.0–10.3)	0/37 (0.0) (0.0–9.5)	5/522 (0.96) (0.3–2.2)

* NE—non-engorged ticks, ** E—engorged ticks; ^1^ χ^2^ = 350.2, *p* < 0.05; ^2^ χ^2^ = 14.85, *p* < 0.05; ^3^ χ^2^ = 214.91, *p* < 0.05.

**Table 2:** Primer sets and *TasI* restriction patterns of BFL1/BFL2 products generated from the *fla* gene fragment of *Borrelia* DNA.

Primer Name	Primer Sequence 5′→3′	Product Size [bp]	Species Reference
BFL1	GCTCAATATAACCAAATGCACATG	442	*B. burgdorferi* s.l.
BFL2	CAAGTCTATTTTGGAAAGCACCTAA		[62]
EHR521	TGTAGGCGGTTCGGTAAGTTAAAG	247	*A. phagocytophilum*
EHR747	GCACTCATCGTTTACAGCGTG		[63]
	*TasI* restriction patterns of BFL1/BFL2 products [bp] [62]		Genospecies
		28-93-321	*B. garinii*
		28-81-89-93-151	*B. afzelii*
		28-89-93-232	*B. burgdorferi* s.s.
